# Association between 1^st^ trimester diet quality & gestational weight gain rate among pregnant women in Dhulikhel, Nepal

**DOI:** 10.1186/s40795-022-00623-7

**Published:** 2022-11-11

**Authors:** Kelly Martin, Diane Rigassio Radler, Joachim Sackey, Cuilin Zhang, Kusum Shrestha, Abha Shrestha, Archana Shrestha, Emily S. Barrett, Shristi Rawal

**Affiliations:** 1 Department of Clinical and Preventive Nutrition Sciences, School of Health Professions, Rutgers the State University of New Jersey, 65 Bergen Street. Room 157, Newark, NJ 07107 USA; 2 Department of Human Ecology, SUNY Oneonta, Oneonta, NY USA; 3 Eunice Kennedy Shriver National Institute of Child Health and Development, Bethesda, MD USA; 4 Department of Public Health, Kathmandu University School of Medical Sciences, Dhulikhel, Nepal; 5 Department of Obstetrics and Gynecology, Dhulikhel Hospital, Dhulikhel, Nepal; 6 Department of Chronic Disease and Epidemiology, Center of Methods for Implementation and Prevention Science, Yale School of Public Health, New Haven, CT USA; 7 Institute for Implementation Science and Health, Kathmandu, Nepal; 8 Department of Biostatistics and Epidemiology, Rutgers School of Public Health, Environmental and Occupational Health Sciences Institute, Rutgers the State University of New Jersey, Piscataway, NJ USA

**Keywords:** Diet quality, Gestational weight gain, Nepal, Pregnancy

## Abstract

**Background:**

Despite promising data from high-income countries, the impact of diet quality on gestational weight gain (GWG) has not been investigated in the context of many low-income countries including Nepal.

**Methods:**

We prospectively examined the association between 1^st^ trimester diet quality and GWG rate among a cohort of singleton pregnant women (*n* = 101; age 25.9 ± 4.1 years) from a community-based periurban hospital in Dhulikhel, Nepal. Diet quality was assessed from the adapted Nepali version of the 21-item PrimeScreen questionnaire in the 1^st^ trimester. The diet quality score is based on consumption frequency of 21 food group components (score ranging 0–42), categorized as healthy (12 groups) versus unhealthy (9 groups), with higher scores indicative of better overall diet quality. The GWG rate was calculated as the measured weight at early-to-mid 3^rd^ trimester (28–35 wks) minus the weight at 2^nd^ trimester (13–25 wks), divided by the number of weeks in between. Linear regression estimated the association between diet quality and GWG rate, adjusting for a priori covariates (i.e. age, education, ethnicity, pre-pregnancy BMI, and nausea/vomiting.)

**Results:**

The mean GWG rate in mid-to-late pregnancy was 0.46 ± 0.2 kg/wk and the mean diet quality score was 23.6 ± 2.5. Based on pre-pregnancy BMI, 49.4% of women had excessive GWG rate, while nearly equal numbers had either adequate GWG or inadequate GWG rate. There was no significant association between diet quality and GWG rate [adjusted β (95% CI) = -0.02 (-0.05, 0.01); *p* = 0.14]. The mean GWG rate was marginally higher (0.57 vs. 0.44 kg/wk; *p* = 0.06) among those with high versus low (2 + servings vs. 0–1 serving/wk) intake of red meat; similar findings were seen when comparing red meat intake between women with excessive versus adequate GWG (Cramer's V = 0.2; *p* = 0.06).

**Conclusions:**

While 1^st^ trimester diet quality is not related to GWG among Nepali women, a high intake of red meat may be a potential risk factor for excessive GWG in this population.

## Background

Historically, maternal undernutrition and inadequate gestational weight gain (GWG) have been the key nutritional concerns and priorities among pregnant women in Nepal [1]. Inadequate GWG is a major contributing factor to low birthweight (BW) and associated risks in the offspring, including increased mortality and morbidity, cognitive deficits, and increased risk for chronic disease as an adult [2]. The rates of underweight (body mass index (BMI) < 18.5 kg/m^2^) among women of childbearing age in Nepal have steadily declined, from 24% in 2006 to 17% in 2016 [3]. However, in recent years, rates of overweight and obesity among women of childbearing age are on the rise in Nepal, increasing from 9% in 2006 to 22% in 2016 [3]. A high pre-pregnancy maternal BMI is associated with a greater risk for excessive GWG during pregnancy, which is also associated with adverse pregnancy outcomes including postpartum weight retention, gestational diabetes, preeclampsia, macrosomia, preterm delivery as well as increased long-term risk of obesity in both mother and the child [4–8]. Identifying modifiable risk factors of suboptimal (either inadequate or excessive) GWG among pregnant women in Nepal is therefore critical in order to inform interventions and prevent subsequent short- and long-term adverse consequences in this population [4, 9–11].

Data from some studies conducted in high income countries show that diet quality is associated with GWG [12–14], whereas others have reported no association [15–20]. Heterogeneity in findings could be attributed to differences in the timing and method of assessing diet quality and GWG across studies. While some studies have defined diet quality based on adherence to identified dietary patterns [14, 21, 22], others have evaluated diet quality based on intake of specific food groups [10, 20, 23]. The timing of dietary assessment during pregnancy also varies across studies, with the majority assessing consumption once, primarily during the 1^st^ or 2^nd^ trimester [10, 12, 13, 15–24]. Most studies have evaluated total GWG as an outcome [10, 12–17, 19, 23, 24], while others have examined GWG by trimester or month of pregnancy [18, 21], and only a few have looked at rate of GWG [22, 25]. What remains consistent across studies is the use of the National Academy of Medicine (NAM) guidelines for adequacy of GWG, though their applicability to populations outside of the United States has not been well established [6].

According to the NAM guidelines, GWG rate is expected to increase linearly in the 2^nd^ and 3^rd^ trimesters with the appropriate rate of weight gain based on pre-pregnancy BMI [6]. GWG rate captures the likely trajectory of total GWG during pregnancy and offers the advantage of accounting for variability in the timing of GWG measures [26]. In addition, GWG rate provides an opportunity for continuous, prospective monitoring of weight status during pregnancy so that interventions may begin earlier if weight targets are surpassed or not achieved [26]. Total GWG, while commonly used, has limitations including a reliance on gestational age, variation in the timing and method of weights obtained for its calculation, and limited inference on the trajectory of GWG across trimesters [27]. GWG rate, on the other hand, can capture dynamic changes in weight gain across different stages or trimesters of pregnancy. For example, rates of GWG are highest during the middle of pregnancy, compared to early and late pregnancy, and more importantly, are associated with neonatal outcomes such as higher birth weight and longer birth length [26].

Despite promising data from high-income countries, the impact of diet quality on GWG has not been investigated in the context of many low-income countries including Nepal. Validated measures of diet quality are often lacking among low-income populations, thus the traditional measure of dietary diversity is commonly used to assess quality of food consumption in these populations [12]. Using a novel brief diet quality assessment tool, our objective was to examine 1^st^ trimester diet quality and its association with GWG rate from the 2^nd^ trimester (13-25 weeks gestation) to the 3^rd^ trimester (28–35 weeks), among a cohort of pregnant women receiving antenatal care (ANC) in a periurban hospital setting in Nepal. We hypothesized that there would be an inverse association between overall diet quality score assessed in the 1st trimester and GWG rate from the 2^nd^ trimester to the 3^rd^ trimester of pregnancy.

## Methods

### Description of study sample

This was a prospective cohort study of 101 pregnant women who were recruited from Dhulikhel Hospital, a community-based tertiary level university hospital of Kathmandu University. Located 20 km outside of Kathmandu, Dhulikhel Hospital has a catchment population of 1.9 million people and delivers approximately 3,500 babies annually. Pregnant women attending the Obstetric Outpatient Department (OPD) at Dhulikhel Hospital for antenatal care (ANC) were recruited for the study between January and December 2019. Pregnant women receiving ANC at Dhulikhel Hospital were eligible for the study if they were age 18 and older, carrying a single fetus, and were ≤ 14 weeks gestation at the time of enrollment. A trained research assistant (RA) collected participant data on diet and other lifestyle and clinical characteristics across multiple ANC visits during pregnancy. Our analytical sample consisted of 85 women who completed all follow-up visits (two per trimester) across the 2^nd^ and 3^rd^ trimesters of pregnancy. The Health Sciences Institutional Review Board of Rutgers University and Kathmandu University both approved the study protocol and each participant provided signed informed consent prior to enrollment.

### 1^st^ trimester diet quality

Diet quality was measured using the Prime Diet Quality Score (PDQS) system and was based on participants’ responses to the modified PrimeScreen questionnaire administered during early pregnancy (5–14 weeks) [28]. The PrimeScreen has been validated for use among adult samples within the United States in the primary care setting [28], among adults at risk for ischemic heart disease [29], and among pregnant women with gestational diabetes mellitus or hypertensive disorders of pregnancy [30]. The 21-item PrimeScreen questionnaire was adapted and translated for the Nepalese diet and has been previously described elsewhere [31]. The modified PrimeScreen was shown to be both a reproducible and valid tool for assessing dietary intake among our pregnant population in Nepal [31].

The PDQS is a measure of overall diet quality obtained from the 21-food group based PrimeScreen questionnaire, which was designed to take into account known relationships between dietary factors and chronic disease [28]. The PDQS is based on consumption frequency of 21 food group components (score ranging 0–42), categorized as healthy (12 groups) versus unhealthy (9 groups), with higher scores indicative of better overall diet quality. Similar to previous studies using this tool, we utilized a scale in which increased consumption of healthy food group components (i.e. fruits, vegetables, whole grains) increases the total score and indicates a higher diet quality (0–1 servings/week = 0 points, 2–3 servings/week = 1 point, 4 + servings/week = 2 points) [29, 30]. The scoring is the same but reversed for the unhealthy food group components (i.e. red meat, processed meat, sugar sweetened beverages, etc.), thus a higher score is given to lower consumption [29, 30]. “The serving sizes represented on the PrimeScreen questionnaire were based on the 2015–2020 Dietary Guidelines for Americans, because a detailed Nepalese dietary guide has not yet been developed. The questionnaire was supplemented with a booklet including colored photographs of various food items, showing their typical serving amount (including amount equivalent to 1 serving) in commonly used sizes of plates, glasses/cups, and bowls in the Nepalese population. The serving amount varied for different food groups (i.e. 1 serving of red meat = 3 oz; 1 serving of fruit = 1 cup or 1 medium size fruit).

In addition to overall diet quality, we also assessed associations of GWG with intake of specific food groups including sugar-sweetened beverages [23], processed meat [32], red meat [32], fruits [33], legumes [34], and dairy intake [33], which were selected based on their previously demonstrated associations with GWG in the literature.

### Outcome

GWG is often expressed as a total weight gain for the entire pregnancy or as an incremental rate in pounds or kilograms per week(6). For the purpose of this study, GWG rate was reported as the rate of weight gain from the 2^nd^ trimester to late pregnancy and was calculated by subtracting the measured weight from the 2^nd^ trimester (13–25 weeks gestation) from the measured weight at the early-to-mid 3^rd^ trimester (28–35 weeks gestation) and dividing this by the number of weeks in between. All weight measures utilized in the study were objectively assessed and were abstracted from the participant’s medical records. Of note, nurse (s), the weighing machine, and the standardized procedures involved in the weight measurements were consistent across all participants, contributing to the reliability of the data. Weight measures were not necessarily obtained in the fasting state and the voiding status was not recorded. Unusually high or low weight measures were checked for possible measurement or recording error. The adequacy of GWG rate was categorized as inadequate, adequate, or excessive based on guidelines from the NAM [6].

NAM recommendations for GWG are based on pre-pregnancy BMI, and in this study 1^st^ trimester weight was used as a proxy for pre-pregnancy BMI [6]. This approach has been deemed a valid alternative due to the known errors in self-reporting pre-pregnancy weight [35], particularly in a population with limited access and means to weigh themselves, such as our population in Nepal [8]. In the 1^st^ trimester, the NAM guidelines state that a woman, regardless of BMI, should gain 0.5 to 2 kg in total [6]. The guidelines then recommend the following weekly GWG for pregnant women in the 2^nd^ and 3^rd^ trimesters: women with underweight status (BMI < 18.5 kg/m^2^) gain 0.44 to 0.58 kg/week, women with normal weight status (18.5–24.9 kg/m^2^) gain 0.35 to 0.50 kg/week, women with overweight status (BMI 25–29.0 kg/m^2^) gain 0.23 to 0.33 kg/week, and women with obesity status (BMI ≥ 30.0 kg/m^2^) gain 0.17 to 0.27 kg/week [6, 36].

### Sociodemographic and clinical characteristics

A structured questionnaire was administered at enrollment and each follow-up visit and collected information on several sociodemographic, lifestyle and clinical characteristics, in addition to data collected from medical record review. For example, data were collected on maternal age, alcohol use before pregnancy, education level, employment status, ethnicity, marital status, smoking during or before pregnancy, depression before pregnancy, height, pre-pregnancy diabetes, nausea/vomiting during pregnancy, pre-pregnancy BMI, and number of prior births.

### Statistical methods

Descriptive statistics are presented as mean (SD) for parametric continuous variables, median (25^th^, 75^th^ percentiles) for non-parametric continuous variables, and frequencies (n, %) for all categorical variables. Raw data was cleaned and checked for normality, and all statistical analyses were performed using IBM SPSS Statistics or Windows, Version 25.0 [37]. The association between diet quality and GWG was examined using a Spearman correlation as the data were not normally distributed. Multiple linear regression estimated the association between diet quality and GWG rate, adjusting for a priori covariates selected based on literature review. These included age, education, ethnicity, pre-pregnancy BMI, smoking status, and nausea/vomiting [4, 10, 12–24]. The mean GWG rate of the sample was also compared by food group intake category using one-way ANOVA analyses. Statistical significance was set at *p* < 0.05.

## Results

### Demographics & clinical characteristics

Maternal characteristics are shown in Table 1.

**Table 1:** Summary statistics of demographic and clinical characteristics (of study participants (*N* = 101)

Characteristic	Mean (SD)
**Age (yrs)**	25.9 (4.1)
**BMI, pre-pregnancy (kg/m**^**2**^**)**	24.3 (3.7)
**Education level (yrs)**	11.8 (3.2)
**ETOH intake, before pregnancy (mL/day)**	119.1 (253.6)
**GWG, rate (kg/wk)**^a^	0.5 (0.2)
**GWG, total (kg)**^a^	9.6 (3.9)
**Height (cm.)**	151.3 (5.9)
**Prime Diet Quality Score (PDQS), overall**	23.6 (2.5)
**n (%)**	
**BMI Category, pre-pregnancy**	
Underweight (BMI < 18.5 kg/m^2^)	4 (4.0)
Normal Weight (BMI 18.5–24.9 kg/m^2^)	56 (55.4)
Overweight (BMI 25.0–29.9 kg/m^2^)	34 (33.7)
Obese (BMI ≥ 30.0 kg/m^2^)	7 (6.9)
**Depression (before pregnancy)**	
Yes	0 (0.0)
No	101 (100.0)
**Diabetes (before pregnancy)**	
Yes	0 (0.0)
No	101 (100.0)
**ETOH Use (before pregnancy**)	
Yes	29 (28.7)
No	72 (71.3)
**Employment**	
Home maker	56 (55.4)
Non-government employee	18 (17.8)
Self-employed	15 (14.9)
Student	7 (6.9)
Government employee	3 (3.0)
Non-paid	2 (2.0)
**Ethnicity**	
Newar	39 (38.6)
Brahmin	22 (21.8)
Magar/Tamang/Rai/Limbu	20 (19.8)
Chetri/Thakuri/Sanyasi	16 (15.8)
Kami/Damai/Sarki/Gaaine/Baadi	4 (4.0)
**Income level (Annual, Nepali Rupees)**	
10,000 – 30,000	3 (3.0)
30,000 – 50,000	58 (57.4)
> 50,000	40 (39.6)
**Marital Status**	
Currently Married	101 (100.0)
Never Married	0 (0.0)
Separated	0 (0.0)
Widowed	0 (0.0)
Cohabitating	0 (0.0)
Refused to answer	0 (0.0)
**Nausea or vomiting** (**1**^**st**^** Trimester)**	
Yes	84 (83.2)
No	17 (16.8)
**Number of Prior Births**	
0	52 (51.5)
1	46 (45.5)
2	3 (3.0)
**Smoking Status**	
Former Smoker	2 (2.0)
Current Smoker	0 (0.0)

*Abbreviations*: *BMI* Body mass index, *kg.* Kilogram, *m* Meters, *n* Number, *N* Number, *%, PDQS* Prime diet quality score, percent, *SD* Standard deviation, *yrs* years

^a^Data obtained in 2^nd^ and 3^rd^ trimester for calculation, *n* = 85

The mean age of the participants was 25.9 ± 4.1 years with a range of 18 to 38 years. On average, participants had completed 11.7 ± 3.2 years of schooling, and approximately half of the sample (55.4%, *n* = 56) were home makers. Newars, who are indigenous to the capital city of Kathmandu and Dhulikhel itself, were the predominant ethnicity represented in the sample (38.6%, *n* = 39). The mean pre-pregnancy BMI of the sample was 24.3 ± 3.7 kg/m^2^ (range 15.8–33.0 kg/m^2^), with a distribution that indicated most women were of normal (55.4%, *n* = 56) or overweight (33.7%, *n* = 34) BMI status prior to pregnancy. All participants were currently married. More than 4 out of 5 participants (*n* = 84, 83.2%) reported nausea or vomiting in the 1^st^ trimester.

### 1^st^ trimester diet quality

**Fig. 1:**
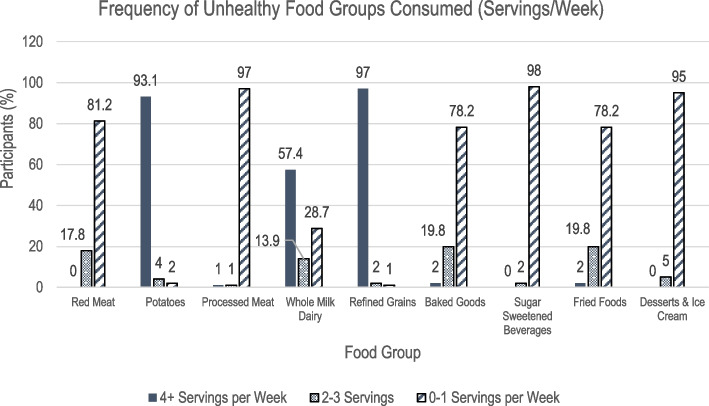
Summary of Prime Screen Data for Unhealthy Food Groups (*N* = 101). Figure 1 describes the frequency of participants’ intake of 9 unhealthy food groups included on the PrimeScreen questionnaire. The healthy food groups with the highest median component scores, indicating a high consumption of these foods were dark green leafy vegetables, citrus fruits, other fruits, and liquid vegetable oils

**Fig. 2:**
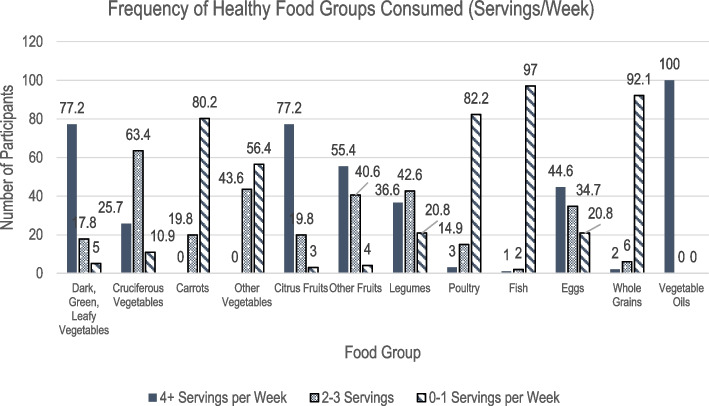
Summary of Prime Screen Data for Healthy Food Groups (*N* = 101). Figure 2 describes the frequency of participants’ intake of 12 healthy food groups included on the PrimeScreen questionnaire. Unhealthy food groups with high consumption included potatoes, whole milk dairy, and refined grains

Overall, the mean PDQS for participants (*N* = 101) was 23.58 ± 2.54 (range 17 to 30) out of a maximum possible score of 42, with a higher score indicative of a higher diet quality. Figures 1 and 2 describe the frequency of participants’ intake of 9 unhealthy and 12 healthy food groups included on the PrimeScreen questionnaire, respectively. The healthy food groups with the highest median component scores, indicating a high consumption of these foods were dark green leafy vegetables, citrus fruits, other fruits, and liquid vegetable oils. Unhealthy food groups with high consumption included potatoes, whole milk dairy, and refined grains. Consumption was notably low for processed meat, desserts, sugar sweetened beverages (SSBs), fish, and whole grains, with more than 95% of participants reporting they consumed only 0–1 servings/week.

### Gestational weight gain

Among our participants, the median incremental GWG rate per week in mid-pregnancy was 0.42 kg per week (range -0.05 to 1.10 kg). Pre-pregnancy BMI and GWG rate were inversely correlated (*r* = -0.21, *p* = 0.05). The adequacy of GWG for each participant was determined by comparing her incremental GWG rate to the NAM recommendations, which categorize GWG rate based on pre-pregnancy BMI [6]. The adequacy of GWG based on pre-pregnancy BMI indicated that most participants had excessive GWG rate (49.4%, *n* = 42), while nearly equivalent numbers of participants had either adequate GWG rate (25.9%, *n* = 22) or inadequate GWG rate (24.7%, *n* = 21). Underweight women were more likely to have inadequate GWG compared to women in other BMI groups (i.e. normal weight, overweight, obesity).

### Determinants of GWG

Pre-pregnancy BMI and GWG rate were inversely correlated (*r* = -0.21, *p* = 0.05). In fact, the highest mean GWG rate (0.50 ± 0.24 kg/wk.) was reported among those with a normal pre-pregnancy BMI (18.5–24.9 kg/m^2^, *n* = 49), while the lowest mean GWG rate (0.39 ± 0.19 kg/wk.) was reported among those with an overweight pre-pregnancy BMI status (25–29.9 kg/m^2^, *n* = 27). Education level was positively and significantly associated with both GWG rate and adequacy of GWG. When comparing mean education level by adequacy of GWG, those with inadequate GWG had fewer total years of education (10.05 ± 3.89 years) compared to those with both adequate GWG (12.43 ± 1.95 years) and excessive GWG (12.36 ± 3.18 years).

### Association between 1^st^ trimester diet quality & GWG

There was no significant association between overall diet quality and GWG rate per week [adjusted β (95% CI) = -0.02 (-0.05, 0.01); *p* = 0.14]. When comparing mean differences in GWG rate by consumption frequency categories of a priori selected food groups, marginally significant associations were observed with red meat intake but no other food groups (sugar-sweetened beverages, processed meat, fruit, legumes, and dairy intake). Specifically, the mean GWG rate was marginally higher (0.57 vs. 0.44 kg/wk; *p* = 0.06) among those with high versus low (2 + servings vs. 0–1 serving/wk) intake of red meat. When comparing red meat intake by GWG category; women with adequate GWG were more likely to have higher intake of red meat than those with inadequate GWG (Cramer's V = 0.2; *p* = 0.06). In regression analyses (Table 2), red meat intake was significantly associated with GWG [adjusted β (95% CI) = 0.13 (0.01, 0.26); *p* = 0.04], even after adjusting for age, education, ethnicity, pre-pregnancy BMI, and nausea/vomiting.

**Table 2:** Association between diet quality and rate of GWG

Variable	Univariate	*P*-value	Multivariate	*P*-value
	**β** (95% CI)		Adjusted^a^** β** (95% CI)	
Age	0.00 (-0.01, 0.02)	0.63		
Education	0.01 (-0.002, 0.03)	0.07		
Pre-pregnancy BMI	-0.01 (-0.02, 0.00)	0.11		
Ethnicity	-0.02 (-0.12, 0.08)	0.73		
Nausea/vomiting	-0.03 (-0.17, 0.11)	0.69		
**Prime Diet Quality Score**	-0.01 (-0.04, 0.02)	0.36	-0.02 (-0.05, 0.01)	0.14
**Red Meat Intake**^**b**^	0.12 (-0.004, 0.25)	0.06	**0.13 (0.01, 0.26)**	**0.04**

*Abbreviations*: *BMI* Body mass index, *CI* Confidence interval, *GWG* Gestational weight gain, *kg* kilograms, *m* meters, *yrs* years

^a^Adjusted for age, education, pre-pregnancy BMI, ethnicity, and nausea/vomiting

^b^Red meat intake comparing 2–3 servings vs. 0–1 serving (none reported 4 + servings for red meat)

## Discussion

In this study we utilized a novel brief dietary questionnaire to characterize 1^st^ trimester diet quality among pregnant women receiving antenatal care in a peri-urban hospital in Nepal, and examined the association between diet quality and GWG in this study sample. The mean diet quality score was 23.58 ± 2.54 out of a maximum possible score of 42, indicating that there is room for improvement in the diet quality of this population. Among healthy food groups, dark green leafy vegetables and fruits were most frequently consumed among our study participants, but consumption was also high for unhealthy food groups such as potatoes and refined grains. Intake of red meat, processed meat, poultry, fish, whole grains, SSBs and desserts were notably low among the sample, with majority reporting only 0–1 serving/week. Based on NAM recommendations [6] nearly half of the participants had excessive GWG rate, while nearly equivalent numbers of participants had either adequate or inadequate GWG rate. Contrary to our hypothesis, there was no significant association between overall diet quality and GWG rate per week. When examining GWG by specific food groups, positive and significant associations were observed between red meat intake and GWG rate per week in our sample of pregnant women.

Studies examining associations between diet quality and GWG in the literature have yielded mixed results, with some reporting significant positive associations [12–14, 25, 32, 33, 38], a few reporting significant inverse associations [13, 25, 32, 38], and others consistent with our findings, reporting null associations [14–21, 23, 24, 34]. Of studies that have reported a significant association between diet quality and GWG, several used dietary diversity or specific dietary pattern measures as a proxy for diet quality [12–14]. Among studies with null findings with overall diet quality, a few studies reported significant associations, both positive and negative, between GWG and the intake of specific food groups or adherence to certain dietary patterns [10, 14, 21–24, 32–34]. Currently, the majority of evidence supporting a link between diet quality and GWG comes from high or middle-income countries. A large Swedish study (*n* = 1,113) [38] for example, observed three to four times higher odds of excessive GWG among women with either poor (OR 3.291, *p* = 0.04) or fair (OR 4.351, *p* = 0.01) diet quality, even after adjusting for several confounding variables including pre-pregnancy BMI. Another large study based in Mexico (*N* = 660) [25], utilized incremental GWG rate per week, and found that both medium (OR 0.77; 95% CI, 0.57–1.04) and high diet quality scores (OR 0.62; 95% CI, 0.41–0.94) were independently protective against excessive GWG. Of interest, this group also found that both medium (OR 0.74; 95% CI, 0.56–0.99) and high diet quality scores (OR 0.63; 95% CI, 0.42–0.95) were protective against inadequate GWG [25]. Of the two studies conducted in low to middle income countries [12, 15] Indonesia and Pakistan respectively, only the latter found a positive association between diet quality and GWG (*N* = 350), but dietary diversity was used as a proxy of dietary quality in that study [12].

In our study, although we did not find a significant association with overall 1^st^ trimester diet quality, we observed that red meat intake was positively and significantly associated with GWG rate even after adjusting for potential confounders including age, education, ethnicity, pre-pregnancy BMI, and nausea/vomiting. Similarly, we found that women with adequate GWG were more likely to have a higher intake of red meat than those with inadequate GWG. In the literature, findings are inconsistent regarding the associations between red meat intake and GWG [32, 39, 40]. Our findings contrast those from a Swiss study which found no association between red meat intake and GWG [40], but are consistent with a study done among pregnant women in Italy, which found that a Western dietary pattern characterized by high intake of red meat was associated with increased GWG [39]. In comparison, a US-based study [32] found that the association between red meat intake and GWG differed by BMI status. Among women with obesity, red and processed meat intake was lower in women with adequate GWG compared to women with either inadequate or excessive GWG [32]. In contrast, they found that among women with overweight BMI status, those who had adequate GWG had higher red meat and processed meat component scores compared to women with excessive GWG [32]. Other studies have noted that an increased adherence to plant-based diets  [24, 34], and diets containing high-quality fats and a low consumption of discretionary foods (i.e. sweets, cakes, soft drinks, and French Fries) may prevent excessive GWG [38], while diets high in fast food [22], and fruit-based drinks [23] may promote excessive GWG. For example, Schlaff et al. [34] found that only the “greens and beans” component score of the Healthy Eating Index-2015 was associated with GWG, specifically at the 35 week timepoint (*p* = 0.04). Yong et al. [33] found that for women with normal weight and overweight/obesity a higher intake of fruit and milk products was associated with higher risk for excessive GWG, particularly in late pregnancy. These findings highlight that pre-pregnancy BMI may play a role in the specific dietary components that protect against suboptimal GWG. Given the small sample size of this study, we were unable to determine whether pre-pregnancy BMI acts as an effect modifier in the association between diet quality/components and GWG.

It is worth noting that the prevalence of excessive GWG rate was relatively high in our study population at 49.4%. Only a few studies have looked at rate of GWG in lower-middle income countries (LMICs). The prevalence of excessive GWG rate in our study did exceed that of a study in Bangladesh where only 19.9% (*n* = 312) of women had excessive GWG rate [41]. The higher prevalence of women with overweight and obesity in our sample (40.6%) could partially explain these findings. Indeed, in the Bangladesh study (*N* = 1569), 19.0% (*n* = 203) of women were overweight or obese prior to pregnancy and more commonly observed to have excessive GWG rate (47.3%) as compared to women who were normal weight (17.4%) or underweight (8.4%) [41]. Similar to what we observed, in a study conducted among a cohort of Mexican women (*N* = 660), 40.9% of the sample were found to have excessive GWG rate during pregnancy [25]. In particular, women with overweight and obese pre-pregnancy BMI status consistently surpassed GWG rate recommendations (58.6% and 53.0%, respectively) [25]. Finally, among a cohort of pregnant Australian women (*N* = 110), excessive GWG rate was reported for 41% of the sample, with the highest proportion of excess (65.0%) observed for those who had overweight BMI [42]. Across the literature, excessive GWG appears to be prevalent among varied populations, and has been reported for nearly 40% of participants in most studies, most utilizing total GWG and not GWG rate as their outcome measure [10, 13, 16–20, 24, 25, 32, 34, 43].

Only a couple of studies have examined rates of inadequate, adequate, and excessive GWG in the Nepalese population, and all were based on total GWG [2, 8]. The mean total GWG in our sample (9.59 kg) was similar or slightly lower than previous studies done among urban sample of pregnant women in Nepal [8, 44]. Notably, when using total GWG measures, 57.6% of women in our study population had inadequate GWG, whereas only 9.4% were found to have excessive GWG, compared to 24.7% and 49.4% respectively when using GWG rate instead. While this is somewhat expected since using GWG rate has been noted for its propensity towards classifying GWG in excess [8, 27], it is worth highlighting that the prevalence of excessive GWG, as measured by GWG rate, is much higher in our sample than expected for a LMIC like Nepal. This suggests that among our study population there may be a rapid weight gain in the middle of pregnancy, with lower weight gain in the early and late stages of pregnancy and low overall weight gain. However, as this is one of the first studies to look at both GWG rate and total GWG in the context of Nepal, we recognize that larger studies in this population are warranted to confirm these findings.

While controversy exists regarding the most valid method for calculating GWG during pregnancy, we utilized GWG rate, as it is well-recognized by NAM, and was also the most appropriate measure based on the data we had available [6, 22, 24, 25]. Incremental GWG rate allowed us to account for the variation in weeks between visits when weight measures were obtained. In addition, we could not use total GWG because the timing of when the final weight measure during pregnancy was obtained varied between patients, and not always available. The use of an incremental GWG rate has been similarly justified by Ancira-Moreno et al. [25] as a way to assess GWG adequacy by trimester and account for variations in the timing of GWG measures. While Gilmore et al. [27] reflect on the fact that GWG rate may not accurately reflect a woman’s trajectory of GWG into late pregnancy or over the entire pregnancy, they note that it is the measure which relies the least on gestational age, thus providing an objective measure of weight between two time points.

This study had several considerable strengths that are worth noting. This was the first study to our knowledge that measured 1^st^ trimester diet quality and its association with GWG rate among pregnant women in Nepal. This study is also one of the first known to examine and characterize baseline pregnancy characteristics in relation to GWG rate among pregnant women in Nepal during pregnancy. The sample was representative of the population with respect to important demographic variables including marital status, employment, and ethnicity, in addition to clinical characteristics such as pre-pregnancy BMI. The nurse (s), the weighing machine, and the standardized procedures involved in the weight measurements were consistent across all participants, contributing to the reliability of the data. However, there are potential limitations that are worth noting. First, participants of the study self-reported their dietary intake information to a trained research assistant, thus the potential for recall bias and data entry error are concerns. Secondly, 1^st^ trimester measured weight was utilized as a proxy for pre-pregnancy weight in the calculation of pre-pregnancy BMI due to the known errors in self-reporting of pre-pregnancy weight, particularly in a population with limited access and means to weigh themselves [8]. First trimester weight is considered to be a valid proxy for pre-pregnancy weight [35] and has also been previously utilized in our study population [8]. However, the potential for misclassifying a participant’s pre-pregnancy BMI based on this proxy measure must be considered. Additionally, weight measures were not necessarily obtained in the fasting state and the voiding status was not recorded. Third, although diet quality was adjusted based on total energy intake (TEI) in a few of the studies reviewed on this topic [10, 13, 16, 17, 22, 24], the PDQS calculated in this research from the PrimeScreen data was unable to determine the TEI at the time of analyses as diet recall information was not available. This limited our ability to estimate nutrient consumption for comparison to existing standards and to adjust for the potential influence of TEI on the outcome of GWG in statistical analyses. Fourth, it is plausible that season could impact the availability of certain food items and thus the diet quality of women included in this study. However, the diets of reproductive-age women in Nepal often remain stable throughout the year with a reliance on carbohydrate-heavy staple foods for the majority of caloric intake [45]. Even when they are in season, fruits and vegetables are often a small percentage of the overall diet, and show little variation by month [45]. Still, in rural areas of Nepal, where there is more reliance on home-produced/grown foods, the impact of season on dietary intake and quality might be greater and should be taken into consideration of future study. Fifth, the observational nature of the study design limits the ability to infer causation, and the short sampling timeframe and the location the sample was drawn from could limit the possibility of certain ethnic and socioeconomic groups being represented in the sample, such as those from rural areas and those that lack transportation or access to healthcare services. We also recognize that this study only examined diet quality in early pregnancy. Future studies may benefit from a repeated diet quality assessment throughout the pregnancy period. National data estimates that access to prenatal care services are improving with nearly 69% of women in Nepal attending at least 4 visits in 2016, up from 50% in 2011 [3]. However, barriers to access still exist, and those with higher income, greater access to transportation, or those that live nearby, or put higher priority on health may have been more willing to participate in the study. Lastly, the NAM recommendations for adequacy of GWG by pre-pregnancy BMI are based on samples in the United States, and thus their actual applicability to Nepal and other countries may be limited [7].

## Conclusion

The mean PDQS in this study indicates that diet quality needs improvement among pregnant women in Nepal, which is similar to findings from other lower-middle income countries across Asia [12, 15], as well as those from upper-middle income and high-income countries around the world (16-19, 24, 32, 34, 38). Results of this study also suggest that pregnant women in Nepal may more frequently experience excessive GWG rate from the 2^nd^ to the 3rd trimester, mirroring similar risks seen among high income countries around the world. While overall 1^st^ trimester diet quality was not related to GWG among Nepali women, we found that a high intake of red meat may be a potential risk factor for excessive GWG in this population. However, as this is one of the first studies to examine and observe this association in the context of Nepal, we recognize that larger studies in this population are warranted to confirm these findings. Further research in this area should continue to examine additional modifiable risk factors of suboptimal (either inadequate or excessive) GWG among pregnant women in Nepal to inform culturally tailored dietary and lifestyle interventions and recommendations in this population.

## Data Availability

The datasets generated and/or analyzed during the current study are not publicly available due to privacy and confidentiality reasons but are available from the corresponding author on reasonable request.

## Abbreviations

ANC: Antenatal care
BMI: Body mass index
BW: Birth weight
GWG: Gestational weight gain
kg: Kilogram
LMICs: Lower-middle income countries
NAM: National Academy of Medicine
OPD: Obstetric Outpatient Department
PDQS: Prime diet quality score
RA: Research assistant
SSBs: Sugar sweetened beverages
TEI: Total energy intake
wks: Weeks
